# Understanding psychiatric institutionalization: a conceptual review

**DOI:** 10.1186/1471-244X-13-169

**Published:** 2013-06-18

**Authors:** Winnie S Chow, Stefan Priebe

**Affiliations:** 1 Unit for Social and Community Psychiatry, Barts and the London School of Medicine and Dentistry, Newham Centre for Mental Health, Queen Mary University of London, London E13 8SP, UK

**Keywords:** Psychiatric Institutionalization, De-institutionalization, Re-institutionalization, Mental Health Care

## Abstract

**Background:**

Since Goffman’s seminal work on psychiatric institutions, deinstitutionalization has become a leading term in the psychiatric debate. It described the process of closure or downsizing of large psychiatric hospitals and the establishment of alternative services in the community. Yet, there is a lack of clarity on what exactly the concept of institutionalization means in present-day psychiatry. This review aims to identify the meaning of psychiatric institutionalization since the early 1960s to present-day.

**Method:**

A conceptual review of institutionalization in psychiatry was conducted. Thematic analysis was used to synthesize the findings.

**Results:**

Four main themes were identified in conceptualizing institutionalization: bricks and mortar of care institutions; policy and legal frameworks regulating care; clinical responsibility and paternalism in clinician-patient relationships; and patients’ adaptive behavior to institutionalized care.

**Conclusions:**

The concept of institutionalization in psychiatry reflects four distinct themes. All themes have some relevance for the contemporary debate on how psychiatric care should develop and on the role of institutional care in psychiatry.

## Background

In the 19^th^ and early 20^th^ century, asylums were the main form of care for patients with severe mental illness (SMI). Illustrating the problematic effects of asylums, Goffman, coined the term “total institution” from a sociological perspective in his seminal work *Asylums*[1]. The concept “total institution” refers to the life of psychiatric patients in institutional settings and originated from his ethnographic fieldwork in 1955–6 in a federal institution of over 7,000 inmates in Washington D.C, United States. His objective at the time was to learn about the social world of the hospital inmates by subjectively experiencing their world. He emphasized that mental hospitals were prison-like institutions although the members had not broken the law. Similarly, he defined ‘psychiatric institutions’ as a closed system apart from the rest of society. He claimed that patients received custodial care and typically lived all aspects of their life in a psychiatric hospital with limited access to the outside world. In a total institution, each phase of the patient’s daily activities was carried out in the immediate company of a large number of other people. All activities were tightly scheduled and the series of performed activities was enforced from the top. Patients’ lives were dictated by institutional routine and isolated from the wider society for an extensive period of time. Goffman further described extensively how inmates underwent a mortification of self, through physical and social abuse, which then lead to the loss of their usual identify. This ‘mortification of self’ involved a process whereby the individual was stripped of their past roles to take on a purely institutional role. In essence, Goffman perceived psychiatric hospitals as establishments that shared the same characteristics as prisons, concentration camps and monasteries and argued that patients were subjected to restriction of freedom, suffered from the stigma of being a psychiatric patient and had their normal social roles taken away.

However, deinstitutionalization of psychiatric patients became widespread as a result of several major factors. Besides the upcoming civil rights movement and the right to receive treatment in the least restrictive environment possible, advances in antipsychotic drugs and alternative care in community enabled the release of patients from mental hospitals. Moreover, the high cost of inpatient mental health care became an increasing financial burden for the developing welfare state. The term ‘deinstitutionalization’ described the process of closure or downsizing of large psychiatric hospitals and the establishment of alternative mental health care in the community. Since deinstitutionalization began in the 1950s, the roles of psychiatric hospitals have changed, and more than half a million long-stay patients have been discharged from psychiatric hospitals in the United States and United Kingdom [2,3]. Psychiatric services in Western countries have moved away from being based on large psychiatric hospitals to community-based care. An extensive body of literature has described de-institutionalization processes, e.g. in Italy [4], Norway [5], Germany [6] and the United Kingdom [7]. The effects of deinstitutionalization vary across countries based on their health care and social welfare systems as well as the specific features of national traditions, socio-cultural context, and the level of available resources [8]. Nevertheless, inpatient psychiatric hospital services are still considered an essential type of care in psychiatry today, as community care may not be suitable for all patients, especially those with acute mental illness and a lack of support. Nearly all patients with severe mental illness are treated mostly in the community yet many people still episodically receive standard inpatient hospital care [9]. It has been argued that the importance of institutionalized care may be rising again regardless of the investment in community mental health services over the past few decades. Supporting this argument, studies suggest that in several countries the provision of institutional care has increased since 1990 [9-12]. Although the number of traditional psychiatric beds continues to fall in most Western countries, a significant increase in forensic psychiatric beds and places in supported housing services in several industrialized countries across Western Europe has been observed. This has been described as ‘re-institutionalization’, whilst others argue that it is a ‘trans-institutionalization’ with patients who would have been long-term hospitalized before de-institutionalization now ending up in different institutions such as residential homes, forensic hospitals and prisons [13].

However, despite the debate on whether the development of mental health care constitutes deinstitutionalization, re-institutionalization or trans-institutionalization, there is little common understanding on how the term ‘institutionalization’ has been conceptualized and understood in the field of psychiatry since the work of Goffman. It is therefore the aim of this paper to review and identify meanings and connotations of institutionalization starting from Goffman’s work on mental hospitals to the present day, focusing mainly within the field of psychiatry and medicine. The objective of this review is not to come up with a new definition but to analyze how the term has been used in the psychiatric literature.

## Methods

A conceptual review of institutionalization in psychiatry was conducted. To synthesize concepts of the phenomenon ‘institutionalization’, the principles of conceptual reviews as described by Lilford et al. [14] were followed. Unlike a standard systematic review, the aim of a conceptual review is not to review all literature but to search widely using various databases and sources; building in safety nets to minimize potential biases (e.g. multidisciplinary study teams) and incorporating some overlap in the various stages of the review process so that the precise direction of the review can be clarified. For the purpose of this paper, the concept of institutionalization described by Erving Goffman in 1961 [1] was selected as the starting point to the diverse and extensive literature because Goffman’s definition of psychiatric hospitals as ‘total institutions’ was influential and still remains strongly in the minds of sociologists, psychiatrists, and service user advocates [15].

To commence, electronic searches were performed and the literature known to the authors was also considered. The databases searched were: Pubmed, Web of Science, PsychINFO, and Scopus as they are widely considered as the most relevant databases for publications in the fields of psychology, psychiatry, and other medical disciplines. For each database, searches were performed using the term “institution* AND severe mentally ill*”, seeking for all literature since Goffman’s work on mental institutions from January 1961 to February 2012. This was later supplemented through additional searches using more psychiatric institutionalization specific terms: (psychiatr* institutionalization AND mental illness) and (mental institution* AND psychiatr*). The titles and abstracts of all identified papers were then reviewed for their relevance. To search widely, the reference lists of all identified relevant papers were also examined to uncover new potential references that were not included in the selected databases. Therefore, although the focus of the review is on the field of psychiatry, some papers also tap into other disciplines such as history, law and sociology if there was a direct link to psychiatry found in the papers by the authors. Full papers were read if necessary to determine their significance before discarding. The concepts uncovered from an initial search of the literature guided further more specific searches around those concepts.

Identified articles were eligible for this study if they met at least one of the following two inclusion criteria: A) mentioning the characteristics, experiences, and/or the functions of adult psychiatric institutions and institutionalization, B) reporting the effect of institutionalization. However, only those papers were included that did not meet any of the following two exclusion criteria: First, papers were excluded if studies about psychiatric hospitals were not based in countries that had experienced major mental health care reforms involving deinstitutionalization during the second half of the 20^th^ century. The reason for excluding such papers is because countries that had not undergone the process of deinstitutionalization at the time may operate on a different organization of mental health care system [16]. Second, papers were excluded if studies focus mostly on psychiatric reforms or the process of deinstitutionalization. Third, papers were also excluded if institutionalization of older adults, children or intellectually disabled were examined, since the review focused on the concept of institutionalization for the core group of patients of working age.

Thematic analysis, a method used for identifying patterns of meaning, was employed to synthesize the findings [17]. Information on the characteristics and functions of psychiatric institutions was extracted from all identified papers and was then analysed in chronological order. This analytic approach was chosen in order to reflect historical trends in the development of psychiatric services. Analysis was regularly reviewed through weekly meetings between WSC and SP, and findings were then presented to the study team and alternative interpretations discussed, in order to minimize any potential biases. Identified categories were then refined, subsumed within existing categories or deleted. The study team comprised a clinical psychiatrist (SD), a research psychologist (RMcC), a psychiatrist who is both a clinician and an academic (SP), and a public health researcher who is also trained as a mental health clinician (WSC). The team drew on their professional experience in various clinical settings, background knowledge of different countries’ health care systems and familiarity with conducting conceptual reviews. The study group was asked to comment on the preliminary themes, the initial draft of this review and to identify any further relevant literature that was not included. The study team’s expertise was utilized to identify patterns as well as to combine related subject matters through discussions. Findings were then grouped based on the underlying concepts which appeared to guide them. The discussions with the study team provided a validity check on the identified themes.

## Results

**Figure 1:**
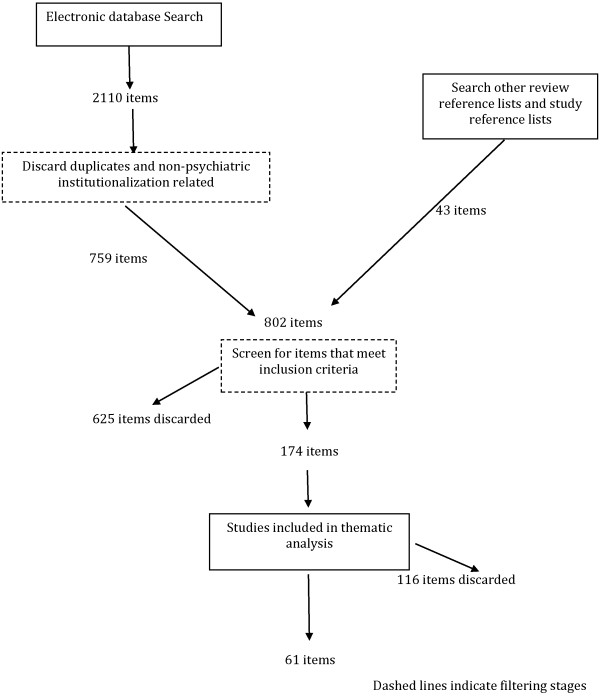
Flow diagram for paper selection.

While this review does not aim for exhaustive searching, a brief summary of the results of the searching protocol is provided as a general understanding of the search process. Figure 1 shows the flow diagram detailing the study retrieval process.

The initial electronic searches produced 2,110 items, which was reduced to 759 after elimination of duplicates and unrelated items. A further 43 items were added from the examination of reference lists. 177 items remained after the elimination of 625 irrelevant materials. Only papers meeting the inclusion criteria were included in the final review (n = 61).

### Overview of papers

Identified publications dated from 1961 to 2012. Data was extracted from 61 papers across eleven Western industrialized countries (Australia, Canada, France, Germany, Italy, Ireland, Netherlands, Sweden, Switzerland, United Kingdom, and United States).

Four main themes were identified. The degree to which these themes have been addressed and specified in the literature varies substantially. They appear to be conceptually distinctive but also to some extent interrelated. The four guiding principles underlying concepts of institutionalization are: a) bricks and mortar of care institutions, b) policy and legal frameworks regulating care, c) clinical responsibility and paternalism in the clinician-patient relationship, and d) patient’s adaptive behavior to institutionalized care. The characteristics of these papers are summarized in Table 1. Each publication sometimes addressed more than one theme.

**Table 1:** **Conceptualization of the term** ‘**Institutionalization**’

**Author(s), year**	**Country**	**Bricks &mortar**	**Policy &legal frameworks**	**Clinical responsibility &paternalism**	**Patients’ adaptive behavior to care**
**1960s**					
Pine & Levinson, 1961 [75]	U.S.A				X
Wing & Brown, 1961 [67]	U.K	X			X
Wing 1962 [63]	U.K				X
Barton, 1966 [69]	U.S.A				X
Gruenberg 1967 [73]	U.S.A				X
Karmel, 1969 [65]	U.S.A				X
**1970s**					
Gomia et al. 1970 [30]	U.S.A		X	X	X
Wing & Brown, 1970 [64]	U.K	X			X
Moos, 1972 [23]	U.S.A	X		X	
Ochberg et al., 1972 [68]	U.S.A				X
Moos, 1973 [24]	U.S.A	X		X	
Rosenhan, 1973 [71]	U.S.A				X
**1980s**					
Johnstone, Owens et al. 1981 [74]	U.K				X
Liberakis, 1981 [70]	Canada	X			X
Goldney, Bowes et al., 1985	Australia			X	
Wasow, 1986 [48]	U.S.A			X	
Talbott & Glick, 1986 [46]	U.S.A			X	
**1990s**					
Myers, Leahy et al., 1990	U.K		X	X	
Wing, 1990 [49]	U.K	X		X	
Curson, et al. 1992 [66]	U.K				X
Abrahamson, 1993	U.K				X
Taj and Sheehan, 1994 [25]	Ireland	X		X	
Prior, 1995 [51]	U.K			X	
Eklund & Hansson, 1997 [47]	Sweden			X	
Breeze, 1998	Sweden			X	
Ford, et al. 1998 [72]	U.K				X
Lutzen, 1998 [61]	Sweden			X	
Owen, Tarantello, et al. 1998 [52]	Australia		X	X	
McCubbin & Cohen, 1999 [59]	UK			X	
Nijman, a Compo et al., 1999 [53]	The Netherlands			X	
**2000s**- **Present**					
Boardman & Hodgson, 2000 [19]	U.K	X			
Quirk & Lelliott, 2001 [56]	U.K			X	
Bowers, Crowhurst et al., 2002 [34]	U.K		X		
Dvoski et al., 2002 [22]	U.S.A			X	
Lewis, 2002 [36]	U.K		X		
Zinkler & Priebe, 2002 [39]	U.K		X		
O’Brien & Cole, 2003 [50]	Australia	X		X	
Salize &Dressing, 2004 [40]	Germany		X		
Priebe, 2004 [18]	U.K			X	
Rittmannsberger et al., 2004 [31]	Europe			X	
Canvin, Bartlett & Pinfold, 2005 [55]	U.K			X	
Karlin & Zeiss, 2006 [28]	U.S.A	X			
Katsakou & Priebe, 2006 [41]	U.K		X		
Killaspy, 2006 [54]	U.K			X	
Quirk, Lelliott & Seale, 2006 [20]	U.K	X	X	X	
De Girolamo et al., 2007	Italy		X	X	
Haglund et al., 2007 [33]	Sweden		X		
McNown Johnson & Rhodes, 2007 [38]	U.S.A	X	X	X	
Sine, 2008 [26]	U.S.A	X			
Johnson, Gilburt et al. 2009 [21]	U.K	X		X	
van der Merwe et al. 2009 [32]	U.K		X	X	X
Priebe, Katsakou et al., 2009 [58]	U.K		X	X	
Coldefy & Curtis, 2010 [29]	France	X			
Katsakou, Bowers et al., 2010 [42]	U.K		X		
Lang et al., 2010 [35]	Germany		X		
Molodynski, Rugkåsa & Burns, 2010 [44]	U.K		X		X
Priebe, Katsakou et al., 2010 [43]	U.K		X		
Sheehan & Burns, 2011 [57]	U.K		X	X	
Lay, Nordt & Roessler,2011 [60]	Switzerland			X	
Georgieva, Mulder & Wierdsma, 2012 [37]	The Netherlands		X		
Reumschussel-Wienert & Crefeld, 2012 [45]	Germany		X		

**Figure 2:**
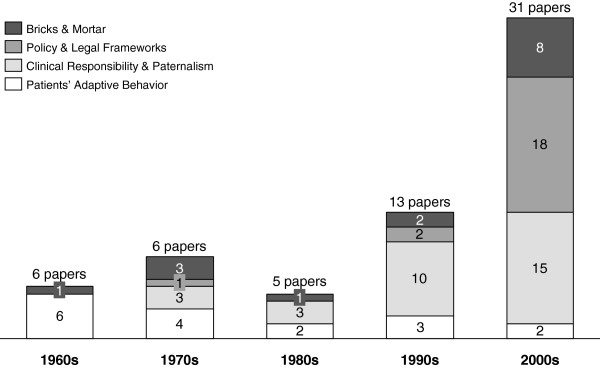
Prevalence of the four identified themes from 1961–2012.

Findings revealed the characteristics and experiences of institutionalization and how the concept evolved and different themes emerged chronologically (see Figure 2). Most of the papers from our review, i.e. 43 out of 61, originated from the last twenty years. Papers from the earlier period focus on recognizing institutionalization as patients’ response to institutional care and the impact institutional care has on patients’ self-concept, while the later papers give emphasis to policy and legal frameworks regulating care and clinical responsibility and paternalism in clinician-patient relationships. In summary, the theme clinical responsibility and paternalism in clinical-patient relationships becomes visible only in recent debates about psychiatric institution, while the concept of institutionalization as bricks and mortar of care institutions has been a part of the conceptualization of institutionalization from its beginnings up until the present day.

### Bricks and mortar of care institutions

Goffman emphasized how psychiatric hospitals were characterized “by the barrier to social intercourse with the outside and to departure that is often built right into the physical plant, such as locked doors, high walls, barbed wire, cliffs, water, forests or moors” [1,15,16]. Such physical elements of ‘bricks and mortar’ are still defined as a key feature of many conventional institutions such as hospitals and residential care amenities in the literature [18]. On the other hand, in a departure from the historical context, the barrier between modern psychiatric in-patient settings and the rest of the world is less clear. Research demonstrates that the expansion of community-based mental health care has reduced the physical boundary and isolation between psychiatric institutions and the outside world [19-21]. It was found, for example, that fencing was picked as the preferred material for the outdoor recreation yards rather than solid walls the a forensic psychiatric unit of the Colorado Mental Health Institute [22].

Similar to Goffman’s notion, a comparable but slightly different way to grasp the concept of a psychiatric institution is by the architectural design of the building [23-26]. The structural design of psychiatric hospitals can play a role in the treatment process but also the safety of the doctors [22,27,28]. Since the early 19th century, the architectural layout of asylums originated from a belief that cure could not occur unless psychiatric patients were isolated from their familiar home environment and put into a suitable “therapeutic space”. Relatedly the term “architectural paternalism” is currently used and the clinical ethics of the architectural design of psychiatric inpatient facilities have been examined [26]. The basis of the ethic of paternalism in the design of psychiatric facilities has also been considered in the context of modern thinking about psychiatric hospitals. Sine argued that the limitation of patients’ rights and autonomy caused by the architectural design of inpatient facilities is legitimate and ethical when it is used to prevent harm and danger [26].

In addition to understanding the physical aspects of psychiatric hospitals as a key aspect of institutionalization, the geographical locations of institutions, i.e. remoteness from local community and cities, has been identified as another characteristic of institutional psychiatric care. In France, Coldefy and Curtis [29] analyzed the geographical locations of specialized psychiatric hospitals from 1800–2000 with a stronger focus on the earlier period. Limitations of classical models of spatial diffusion, the processes of the conservation and transformation of geographical spatial structures, were found although not consistent with all the different phases of development of psychiatric institutions. The developmental process of these psychiatric hospitals seems to be associated with the national policies, social representations, and medicalization of care of mental illness, urbanization and economic growth. The authors therefore suggested that a political ecology approach, a model that takes into account the relationship between political, economic and social factors with environmental issues and changes, might be more appropriate to understand the vast development of French psychiatric care [29].

As Figure 2 reveals, the theme of bricks and mortar has constantly been in part discussed in the literature over the time period covered in this review. However, relatively few papers have focused on this theme prominently compared to others. The narrow focus may have been triggered by the deinstitutionalization movement and the negative perception of the institutions as dehumanizing and damaging for the mentally ill. Despite the negative connotation people have formed about institutions, it appears that mental health professionals have always been concerned about this aspect of mental health care as it is an underlying principle of moral therapy – it defines the physical place where care is provided and where treatment is give to patients and thus has always been part of the debate.

### Policy and legal frameworks regulating care

Before the radical shift from large psychiatric hospitals to community-based services, the physical building of large mental hospitals defined institutional care [1]. However after the deinstitutionalization movement, institutional care has also been conceptualized in terms of the policies and legal framework of the relevant institutions and national legislation that limit the patients’ autonomy. Although there has been a tendency to open wards up and allow patients free movement, many psychiatric hospitals still operate to some extent as a safeguarding system, and a considerable amount of care is still provided behind locked doors [30-32]. For instance, large numbers of Swedish inpatient psychiatric wards are locked [33] and 22 out of 87 acute wards in London were locked permanently according to a study in 2002 [34]. This occurs despite evidence from a German study that a closed entrance door to an acute psychiatric ward did not reduce absconding [35]. In an ethnographic study of three acute wards in London, Quirk and colleagues found that entrance doors may also be locked temporarily to prevent patients from escaping while some patients might be required to transfer to a locked, intensive care unit [20]. On wards that have more of a permeable nature, instead of locking patients up, an alternative method has been employed to manage the risk of patients running away or self-harming – a staff member is appointed to observe the patient closely at all times. Besides placing a patient in a locked care unit, seclusion, restraint and sedation are also identified as interventions to monitor and control the high-risk and potentially dangerous behaviors of a patient who is experiencing a severe psychotic episode [36,37].

Restriction of freedom is still often associated with psychiatric institutionalization and hospital treatment although modern psychiatric wards and hospitals have been found to be ‘permeable’ [20]. Similar to Goffman’s interpretation of psychiatric hospitals, McNown Johnson & Rhodes characterized psychiatric institutions as establishments where their residents have little or no choice about their participation in activities, and have little say about how they are being treated [38]. Admitted residents are not allowed to leave the psychiatric institution without being officially released or discharged. From this perspective, patients’ freedom of movement is restricted and the functions of psychiatric institutions are similar to a security guard.

Besides exploring locked facilities as one type of psychiatric treatment model, legislation has also been set up for the practice of involuntary placement or treatment of people with mental illness. The mental health law and legal framework for involuntary placement or treatment varies across Europe. Significant numbers of patients in Europe are involuntarily admitted to psychiatric hospital units [39]. Frequencies of compulsory admission were found to vary across the European Union [40]. However, law and practice does not always coincide. Katsakou and Priebe [41] found that many patients feel retrospectively that the involuntary admission was justified while another study revealed a significant proportion of formally voluntary patients feel coerced [42]. The variation across countries might be related to differences in legislation between countries [43]. The differences between the legislation and patients’ view of mandatory treatment often lead to question whether admission was right or not. Therefore, it is critical to regulate any psychiatry practice that limits the autonomy of an individual.

Restriction of freedom of choice and social integration of patients with mental illness may also occur in community psychiatric treatment settings. In England and Wales, the Mental Health Act 1983, which was amended in 2007 considerably, allows individuals with a mental disorder to be admitted to hospital, detained or treated against their will for both their own health and safety or for the protection of the general public. Compulsory community treatment was introduced as one of the amendment to the Mental Health Act 1983. Molodynki, Rugkåsa and Burns [44] suggest that the Mental Health Act has increased the capacity for compulsion in the community and is reflected in the recent changes in service provision, although the evidence base is relatively small. In Germany, the advantages and disadvantages of closed psychiatric homes in Berlin were discussed recently in a debate paper [45]. Reumschuseel-Wienert argued for closed psychiatric homes because community psychiatric facilities are not capable of providing sufficient care for patients with severe limitations, such as a lack of insight into their illness, an inability to regulate or control their emotions, or to structure their time and the organization of their self-care. Crefeld, on the other hand, suggested that it is not unknown that patients with severe mental impairments often need help to cope with everyday life. He claimed that it is difficult to provide person-centered treatment in closed psychiatric homes because this form of care generally offers all residents the same consistent care package regardless of whether the individual residents need it or not.

As the numbers in Figure 2 show, the attention to the theme of policy and legal framework emerged after the year 2000. Before this, little attention was paid to this aspect of institutionalization. This may be because most mentally ill people are no longer treated in large mental hospitals in remote areas as a result of the changing pattern of mental health care – the closure of large mental hospitals, the decline of psychiatric hospital beds, short stay admissions and the development of care in community. Therefore the emphasis has then shifted towards more on the legal aspect, such as the rise of compulsory treatments [40].

### Clinical responsibility and paternalism in clinician-patient relationships

Institutional care can also be characterised by the service organization and the responsibility that mental health professionals have for patients. Besides safekeeping the patients, many treatment and care elements such as shelter and protection are also provided on modern inpatient hospital wards [46]. Inpatient treatment for instance offers the chronic mentally ill patients, whose symptoms cannot be controlled in an outpatient program a structure in which treatment can effectively control their symptoms. For instance, antipsychotic medication has been considered as a primary inpatient treatment modality. It has been seen as helpful and effective in suppressing psychotic symptoms in the hospital, but also as potentially hindering community adaptation on discharge. For this reason, Talbott and Glick argue it is essential to reduce medication at some point after discharge [46].

While many mental health professionals perceive psychiatric institutions as a treatment model that is isolating the mentally ill, in the late 1990s, the treatment environment provided by inpatient wards has been considered potentially beneficial for patients [47]. Linked to this, psychiatric institutionalization has been seen as providing protection and care to patients who are chronically mentally ill [46,48-51]. It has been highlighted that even the best community care does not offer enough care and protection for the many chronically mentally ill and the need for sanctuary and asylum can only be provided as an institution of some kind [48]. Wasow claimed that institutionalization does not necessarily cause dependency; rather it provides a permanent, structured, supervised housing for the chronically mentally ill [48]. In addition, institutional care protects this vulnerable population from the prejudice and the hostility that they might experience in the larger society. Samuel, a typical case of a single patient, who spent 36 years in a large mental hospital in Northern Ireland, was reported as an example of a patient utilizing the hospital as a lodging house. Meanwhile he did odd jobs such as gardening for his fellow churchgoers and went to church regularly in his last ten years [51]. He had been an involuntary patient for the first 25 years of his stay and then refused to be discharged from the institution because he was happy with his life at the time.

However, despite the fact that the main purpose of psychiatric institutions is to provide a stable environment to facilitate the treatment process so that patients’ psychotic symptoms could be reduced, nevertheless patients’ safety and wellbeing are threatened by violence from patients on inpatient psychiatric wards [52,53]. Nijman and his associates claimed that the hospital’s environment inescapably introduces stressors on the patient. The violent behaviour by patients with psychotic disorders on the wards is exacerbated by some negative forms of environmental and interpersonal stimulation such as the disorganization of a crowded psychiatric ward, noise [22], the lack of interesting activities, and/or problematic communication with staff members.

A more recent way to understand institutionalization in psychiatry is in terms of the relationship between staff members and patients. In the present day, psychiatric care does not rely solely on hospital facilities. As a result of the large reduction of psychiatric hospital beds and the re-focus of institutionalized care to community treatment, more people with severe mental illnesses are treated in community-based settings [9,54]. There are several residential alternatives although they cannot be considered as an optimal option for all patients to acute inpatient psychiatric services [19].

To conceptualize institutionalization purely based on the length of hospital stay within bricks and mortar, locked up hospitals or basing it on the change of patients’ identity and social position prior/after to admission might not reflect the practice of institutionalization in contemporary psychiatric institutions. For example, institutions can be understood as a web of people, ideas and practical/potential power in our contemporary society [55]. Moreover, patient-nurse relationships are recognized as an essential aspect of therapeutic psychiatric in-patient care [56]. A cross-sectional cohort study of the association between perceived coercion and therapeutic relationship by Sheehan and Burns [57] concluded that “hospitalization, even when voluntary, was viewed as more coercive when patients rated their relationship with the admitting clinician negatively”. Moreover, patients’ perception of their treatment engagement matters. Priebe and his team found in an observational prospective study that involuntarily admitted patients with initial satisfaction with treatment were associated with more positive long-term outcomes [58]. They concluded it is important for clinicians to consider patients initial views as a relevant indicator for their long-term prognosis of involuntarily admitted patients. Moreover, “institutions do not necessarily have walls” [18]. Staff and patients in community treatment teams such as assertive outreach engage in an obligatory close relationship, as the aim of community services is to provide treatment to people who do not seek it themselves. Whether services are being provided on wards or in the community, these intense relations between staff and patients may also define institutionalized care, particularly if the social interaction among members of an institution is mandatory as a result of involuntary admission.

The relationships between the clinical staff and patients as well as among patients themselves are unequal in terms of social power. For instance, on wards very few admitted patients have “privileges” in terms of the allocation of preferred accommodation, access to social facilities, activity, or extra food [30]. Members of staff are required to keep an eye on the admitted patients on a regular basis to ensure patients are not in any danger. Clinical staff, particularly psychiatrists, have authority but also responsibility for patients’ safety [59]. Patients’ right to autonomy is nonetheless usually restricted by staff in psychiatric inpatient wards for their wellbeing. It has been found that staff members behave more paternalistically towards patients within highly formalized institutions, but are more in agreement with patients in less formal ward environments [20]. Also, depending on the culture of the wards or mental hospitals, patients can either be motivated to speak or made quiet by staff [20].

Relatedly, the paternalistic relationships between staff and patients are also shown through the use of coercion. A variety of forms of coercion (informal or formal) is frequently practiced by clinical staff to ensure medication adherence [60]. The openness between a clinician and his or her patient/client could change depending on the social culture of the institution such as treatment design and the mental health as well as the legal status of the patient (i.e. voluntary versus involuntary). In a mixed methods study, Katsakou and associates [40] identified that roughly one third of the voluntary patients felt coerced into admission and half of them continued to feel coerced into treatment a month later. Patients felt less coerced if their satisfaction with inpatient hospital treatment also increased. Yet the usage of coercion is often justified in mental health settings on the notion that patient’s health condition hinders his or her ability to make a sound decision [61,62]. Formal coercive treatment outside hospitals such as community treatment orders are also commonly accepted and practiced [43].

The theme of clinical responsibility and paternalism emerged in the 1970s but as the numbers in Figure 2 suggest, attention to this theme increased substantially in the 1990s. In this decade, the majority of the identified papers included this theme. This may be explained by the general debate during this time frame on how to best care for patients or serve those service users most in need – the act for balancing the rights of the patients and the responsibilities of the clinical professionals.

### Patients’ adaptive behaviour to institutionalized care

Institutionalization in psychiatry can also be characterised by symptoms exhibited by patients in response to being treated in an institution, i.e. the patients’ adaptive behaviour to care. Institutionalism was a term adopted by Wing [63] to describe a trend observed during a study of the long -stay male patients of two large hospitals in 1950s in England, which he later on also termed ‘social withdrawal’ [64]. Initially it was recognized as a syndrome in inpatient psychiatric facilities, and is now used to describe a set of maladaptive behaviours that are induced by the tensions of living in any institution [37,64-66]. Wing and Brown [64] defined institutionalism as the association between the poverty of the physical environment and severity of primary symptoms of the illness and secondary disabilities that are not part of the illness itself, and identified three variables that increase the damaging effect: the social pressures that stem from an institution, the length of time that the resident was exposed to these pressures, and the level of predisposition that the resident brought [63,67].

Wing & Brown [64,67] studied the impact of institutionalized care on patients with severe mental illnesses. The objective was to test the notion that there is an association between the social conditions of psychiatric hospitals and the clinical state of the patients. Wing and Brown found that patients with schizophrenia had fewer negative symptoms when they were treated in hospitals with richer social environments and opportunities. In addition, these patients showed distinctly fewer disturbances in verbal and social behaviour. In contrast, patients with the least social interaction, fewest activities to take part in, and the least access to the outside world were the most unwell.

Patients who reside in any institutional setting such as psychiatric hospitals or prisons are often socially isolated or have limited access to the outside world. In other words, individuals in institutions may lose independence and responsibility, to the point that once they return to life outside of the institution, they are often unable to manage everyday demands. A number of authors preferred the term “institutionalism” for this phenomenon [68], while Barton [69] argued the term “institutional neurosis” is more adequate to refer to the disability in social and life skills as a result of adaptation to the demands of an institution. He also stated that the term “institutional” does not indicate that institutions are the only cause of such disability, and that the behaviour was only first recognized in institutions. Institutionalism, defined as “the impoverishment of feelings, thoughts, initiative and social activity” may be found among patients in boarding homes and some premorbid features of patients, i.e. low intelligence, poor education and disability in hearing, speech, locomotion and manual dexterity, may make them more susceptible to institutionalism than others [70].

Alternatively, depersonalization and the loss of one’s identity have been suggested as key features of institutionalism [1,71]. Institutional environments can be perceived as humiliating, and admissions to acute psychiatric wards can be stigmatizing and non-therapeutic [72]. Many inpatients upon admission adapt to their environment intrinsically, particularly those who live for prolonged periods in restricted environments. They become dependent on receiving care from services, lose their confidence to make decisions and consequently become institutionalized.

Similarly, Gruenberg linked institutionalization to “social breakdown syndrome” (SBS) [73]. SBS can be characterized as the loss of normal role functioning with a varying degree of exclusion from typical family or community roles. The features are similar to the negative symptoms of schizophrenia. SBS can be the by-product of any treatment that removes the patient from his or her regular social environment (i.e. long-term hospitalization or “overprotection” excessively on the part of clinical staff and/or family members). The author claimed that there are seven stages of SBS and compared the last stage, ‘identification with the sick’, with Goffman’s last mode “conversion”. He argued that in such a stage a patient accepts the status of the chronic sick role and identifies with the other sick patients around him.

However, on the other hand, not all long-stay patients are affected negatively by psychiatric institutions. No difference in terms of cognitive deficits was found in a study comparing schizophrenic in-patients and out-patients, when age and duration of illness were accounted for [74]. Pine and Levinson [75] argued the relation of a patient to a mental hospital can be described as “patienthood” and claimed that those patients who become resident in a mental hospital voluntarily are like college students. Although being a patient in a mental hospital consists of punishment and stigma similar to being incarcerated in prison, the admission can also be seen as an opportunity for personal growth and social advancement like going away to university particularly when patients can adapt and adjust to their physical environment, staff and other admitted patients.

The theme of patient’s adaptive behaviour has been part of the literature throughout the whole period covered by this review. However, after the 1960s, only a small share of the identified papers covers this theme. The significant reduced emphasis on patient’s adaptive behaviour as a theme over time might have been introduced by the change in the mental health care model, from providing care in institutions in remote area to care in the community. Patients now are living and being cared for in new settings in the community.

## Discussion

Four different meanings of how ‘institutionalization’ in psychiatry is conceptualized were identified from sixty-one papers across eleven different countries, i.e. bricks and mortar of care institutions, policy and legal frameworks regulating care, clinical responsibility and paternalism in clinician-patient relationships, and patients’ adaptive behavior to institutionalized care. These four identified connotations of how the term has been used in literature are conceptually distinct, but appear to overlap. Seventeen papers contained more than one of the four themes which may illustrate the complexity of the concept of institutionalization.

The conceptualization of institutionalization in psychiatry appears to have changed over time along with the changes in the provision of mental health care. Prior to the movement of deinstitutionalization, old-style mental hospitals functioned merely as a custodial care model and thus the perspective of bricks and mortar prevailed. The term ‘deinstitutionalization’ describes the process of downsizing and closing large hospitals accompanied by the establishment of alternative community-based mental health services [76,77]. As a result of the process of deinstitutionalization, many long-term hospitalized patients then were discharged into the community. It was found that the discharged patients experienced a higher quality of life compared to the hospitalized patients. Examples for such research are the studies of the Team for the Assessment of Psychiatric Services (TAPS) in North London [78] and the Berlin De-Institutionalization study [79]. Discharged patients reported better satisfaction with their living conditions and had acquired friends and confidants. In addition, they gained domestic and community living skills, although no change was found in the patients’ clinical state or in their problems of social behavior. In modern psychiatry, however, the term ‘institutionalization’ goes beyond bricks and mortar as the functions of mental hospitals have changed. While in modern psychiatric hospitals less emphasis is put on institutionalizing patients with bricks and mortar, institutionalization is rather displayed in terms of policy and legal framework, in terms of clinical responsibility and paternalism or understood as patients’ response to institutional care. Although institutional organization and clinical responsibility aim to provide a structured and safe environment to facilitate the treatment process and to help monitor patients, they can also unintentionally institutionalize patients. Clinical paternalism can reinforce patients’ dependency on services, for instance, in the case of mandatory relationships between staff and patients where staff offer clinical paternalism- with the best intentions- to help patients manage their symptoms and life. Patients’ mental capacity to consent to treatment may also have to be considered in this context, but has so far received little attention in the literature on psychiatric institutionalization.

If paternalistic relationships between staff and patients reflect institutionalization, then institutionalization must not necessarily occur in a physical facility such as a mental hospital, but patients may also be subjected to being institutionalized in supported housing or supervised residential facilities with around the clock staffing as well as other alternative institutions in community settings (i.e. forensic hospitals). In addition, if institutionalization is conceptualized as patients’ response or adaptive behavior to services, then specialized community care such as assertive outreach could also be seen as a form of institutionalization due to the limited patient autonomy and their dependency on the intensive comprehensive care. Mental health patients residing in highly structured environments of community-based sheltered-care facilities can exhibit a distinct pattern of dependency [80]. Assertive Outreach (AO) has already been criticized for being paternalistic and coercive [44]. Service users of AO teams live, work and socialize in the community as “free individuals” yet they remain subject to rules and restrictions as if contained in old fashioned asylums [18]. Furthermore, patients who are legally mandated to receive treatment such as compulsory treatment in hospital or community might also be at risk of being institutionalized even though some argue involuntary psychiatric care helps to reduce symptoms, manage illness and re-establish a person’s ability to make autonomous decisions.

In conclusion, despite modern psychiatric services continuing to reflect the trend of deinstitutionalization with the closure of large mental hospitals, reduction of psychiatric hospital beds and the discharge of long-stay hospitalized patients into community, the findings of this review suggest that institutionalization can still manifest in alternative forms of community-based institutional settings. Therefore, there is a risk that mental health patients might also be subjected to new forms of institutionalization in community-based services, as conceptualized in the four identified themes. Although the establishment of community care aimed to promote patients’ autonomy and to provide care and treatment on a ‘partnership and consensual basis’ as much as possible, this review shows that institutionalization can still manifest in modern psychiatry similar to the old -style mental hospitals (asylums) beyond the traditional bricks and mortar hospital settings [81,82]. While patients may prefer community-based care to institutional ones, there is still a risk of subjecting mental health patients to institutionalization on psychiatric acute wards in general hospitals or new forms of residential facilities in community settings.

The results of this review can be related to critiques of Goffman’s notion of the mental institution [20,37,83,84] namely that the earlier conceptualizations of institutionalization are limiting and can no longer be applicable in today’s context. The traditional conceptualization of institutionalization reinforces mainly a restrictive understanding of institutionalization as taking place in institutions, where patients are only the sufferers of the treatment process and have limited autonomy and are completely isolated from the outside world. Townsend [82] concluded in his review that studies from 1959 to 1975 support the idea that institutionalization involves patients accepting institutional life and developing a lack of desire to leave after a long stay in mental institutions. More recently, Quirk and his associates [20,56] found that ‘permeable institutions’ provide a better representation of the reality of everyday life in modern 'bricks and mortar' psychiatric institutions.

### Strengths and limitations

One of the strengths of the review is that it considered literature from different disciplines and countries. Several main databases in the field were searched with broad search terms to avoid missing any major debates or discussions in the literature. In addition, the study team’s expertise in psychiatry, psychology and public health were utilized to identify patterns, combine related subject matters and minimize potential biases.

The review also has a number of limitations. Since the aim of this review is to search widely, relevant articles may have been missed but also literature that does not contain the search terms of this review explicitly. Also, conference presentations or grey literature were not included. The replicability of the review is limited given that establishing what information is relevant was based mainly on the individuals who are conducting the review. Finally, due to the focus of this review on the field of psychiatry, it has been beyond its scope to appraise how the term institutionalization is used in other disciplines. Consequently, a wide body of literature in the social sciences such as those examining the institutionalization of inmates, juvenile offenders or children in institutional care has been excluded.

## Conclusions

The findings of the review emphasize that the term ‘institutionalization’ in modern psychiatry goes beyond definitions based on bricks and mortar, but rather includes ideas about staff’s responsibility, and policy and legal framework. Based on the traditional perspective of institutionalization, new services in the community can be seen as part of de-institutionalization. From the contemporary viewpoint however, one could argue that services such as supervised supported housing or assertive outreach may be a new form of institutional care since the movement of de-institutionalization.

The identified themes provide a preliminary framework for investigating and analyzing all care institutions in modern psychiatry, but do not constitute a coherent theoretical model of institutionalization. In this conceptual review, we neither started with an overarching theory nor developed one, but showed how institutionalization is understood in the field of psychiatry. This review highlights a number of fundamental elements to consider in further examination of the current and future development of institutional psychiatric care.

The findings have implications for further empirical research on (de)institutionalization. Tallying and analyzing the number of beds and places in mental health institutions facilitates health systems to examine and monitor their current state and future direction of mental health care institutions methodically. Moreover, provision of bed numbers in institutional settings can provide an indication of the trend of (de)institutionalization from a bricks and mortar perspective. Surprisingly, however, such data is difficult to obtain and clearer definitions and reliable sources are still required to investigate trends over time and even more so to compare internationally [84,85]. Thus more research is needed to fully understand the international development of mental health institutions. Yet, as this review showed, bed numbers in mental health institutions will not capture all of the different but interrelated aspects of psychiatric institutionalization. Therefore, quantitative research on trend(s) of (de)institutionalization should also include the development of other forms of psychiatric care, e.g. supervised supported housing, on the agenda. Also here, the availability of international data and identifying common definitions will be a major challenge. In spite of this, going beyond the bricks and mortar perspective in empirical research on (de)institutionalization will provide a valuable starting point for further quantitative and qualitative investigation of the underlying reasons for the changes in the provision of institutionalized mental health care.

To fully understand if and how alternative forms of psychiatric care can bring about a different form of institutionalization, it will be essential and worthwhile to adhere to the theme of patients’ adaptive behavior to care. This will shed light for a quantifiable approach to measure institutionalization in different contexts. Once more such research will lead to a thorough understanding of how patients respond or adapt to the different type of institutional psychiatric treatment, which will allow researchers to explore if a process of ‘psychiatric institutionalization’ is also apparent in other specialized modern forms of community mental health care model such as assertive outreach teams, early intervention teams and or crisis resolution terms.

From a public health perspective, institutionalization as policy and legal frameworks regulating care has been increasingly examined in the recent past as the rate of involuntary admission of people with mental illness has increased over the years [40]. In taking a step forward, it will be fruitful to also include clinical paternalism and responsibility on the research agenda when looking at the effects of psychiatric institutionalization. For example, Sheehan and Burns’ findings [57] indicated that high levels of perceived coercion are significantly associated with involuntary admission and a poor rating of therapeutic relationship. Voluntary hospitalization was seen as more coercive when patients rated their relationship with the admitting clinician negatively. The mental health field needs to assess the influence that paternalistic relationships and the unequal power relation between clinical staff and patient could have on the patient’s health condition and autonomy. More importantly, more empirical studies are needed to examine the benefits and harms of using informal coercion and practicing compulsory community psychiatric care.

In summary, the term ‘institutionalization’ is an evolving concept. Hence, further investigation of any of the four identified distinct but related themes will help develop a fuller understanding of (de)institutionalization and thus aid in clarifying the direction of mental health care in continuous discussions and future debates.

## Competing interests

The authors declare that they have no conflicts of interest.

## Authors’ contributions

WSC and SP were involved in the conception and design of the review. WSC conducted the literature search, interpreted data and drafted the manuscript. Both authors read and approved the final manuscript.

## Pre-publication history

The pre-publication history for this paper can be accessed here:

http://www.biomedcentral.com/1471-244X/13/169/prepub
